# Positive Rotational Internal Limiting Membrane Covering Technique for Large Idiopathic Macular Holes

**DOI:** 10.1155/joph/9081680

**Published:** 2026-01-06

**Authors:** Huiyu Xi, Yingying Song, Yewen Ni, Yumei Cao, Tianyu Zhu, Wei Fan, Haiyang Liu

**Affiliations:** ^1^ The Affiliated Xuzhou Municipal Hospital of Xuzhou Medical University, Xuzhou, China; ^2^ Department of Ophthalmology, Xuzhou First People’s Hospital, Xuzhou, China; ^3^ Eye Disease Prevention and Treatment Institute of Xuzhou, Xuzhou, China; ^4^ Department of Ophthalmology, Kaifeng Central Hospital, Kaifeng Eye Hospital, Kaifeng, China

**Keywords:** large idiopathic macular hole, positive internal limiting membrane flap, rotational covering technique

## Abstract

**Purpose:**

This article aims to determine the efficacy of a modified technique by a positive rotational internal limiting membrane (ILM) covering for the treatment of large idiopathic macular holes (IMHs).

**Methods:**

A prospective analysis was conducted on a cohort of 13 patients with large IMH (> 400 μm). The ILM was dissected in approximately one diameter width of the optic disc from the inferior and temporal sides. Subsequently, a pedicled ILM flap connected to the optic disc was grasped and peeled from the superior edge of the residual ILM with a width of at least 2 papilla diameters (PDs). The pedicled ILM flap was then directly rotated to cover the IMH in a positive way. Some peeled‐off ILM samples were collected for scanning electron microscopy (SEM) examinations. During each follow‐up, best‐corrected visual acuity (BCVA), SD‐OCT scans, and M‐CHARTS were performed.

**Results:**

The study successfully detected IMH closure in all cases. The mean BCVA (logMAR) showed a decrease from 1.18 ± 0.209 to 0.58 ± 0.202 (*p* < 0.001). Postoperatively, there was a significant reduction in the diameter of the ellipsoid zone (EZ) and external limiting membrane (ELM) defects compared to preoperative values (*p* < 0.001). Additionally, the defect size decreased further at the 3‐month follow‐up compared to the 1‐month follow‐up. Both horizontal and vertical deformations postoperatively showed significant improvements (*p* = 0.001, *p* < 0.001). At the final follow‐up, 7 eyes exhibited U‐shaped closure while 6 eyes showed V‐shaped closure.

**Conclusion:**

This technique is an effective treatment for larger IMHs. This technique has the potential to improve vision outcomes.

**Trial Registration:** Chinese Clinical Trial Registry (ChiCTR): ChiCTR2300068411

## 1. Introduction

Pars plana vitrectomy (PPV) combined with internal limiting membrane (ILM) peeling and gas tamponade was first introduced as a treatment for MHs by Kelly and Wendel in 1991 [1]. It has been widely accepted as the standard surgery for idiopathic macular holes (IMHs) due to the high closure rate, especially for those with a size less than 400 μm. However, for IMHs larger than 400 μm, the closure rate is lower. The application of the ILM covering technique can improve the closure rate and lead to a better functional prognosis for large IMHs. Studies have demonstrated that the ILM flap can act as a scaffold for the proliferation of glial cells along with remaining Müller cells [2]. The inverted ILM flap technique, first reported by Michalewska, is one of the most widely studied surgical procedures [3]. However, it has been reported that the inverted ILM flap technique may not effectively contribute to the reconstruction of the outer retinal layers and vision restoration in cases of large IMH [4], as it provides a smooth surface for the IMH covering and results in fewer Müller cells [5]. Therefore, a modified technique involving a positive pedicled ILM flap that is created and rotated directly to cover the IMH, followed by the use of autologous blood clot (ABC) to fasten the flap, may be beneficial for reconstructing the microstructure of large IMHs and improving visual outcomes.

## 2. Methods

A prospective and consecutive study was conducted at Xuzhou First People’s Hospital from May 2022 to August 2023. Twelve patients with large IMHs (> 400 μm) underwent vitrectomy using a new technique. Presurgery, we recorded the best‐corrected visual acuity (BCVA) and basic eye conditions. Spectral domain optical coherence tomography (SD‐OCT) was used to verify and measure the IMHs. Metamorphopsia changes were evaluated using M‐CHARTS before and after surgery, specifically using the two‐line type due to central scotoma in IMHs. Detailed examinations, including BCVA, intraocular pressure (IOP), M‐CHART, slit‐lamp examination, fundus examination, and foveal microstructure using SD‐OCT, were conducted before and after surgery. Additionally, eight patients underwent multifocal electroretinogram (mf‐ERG). Patients with highly myopic and other ocular disorders, except mild‐to‐moderate cataracts, were excluded. The study adhered to the principles of the Declaration of Helsinki and was approved by the Ethics Committee of Xuzhou First People’s Hospital. The surgical procedure, detailed in a supporting video (see video [here] and Figure 1), involved a standard 23‐G PPV (Alcon, Constellation) under retrobulbar anesthesia by a single experienced surgeon (H.L.). All patients had cataracts and underwent phacoemulsification with intraocular lens implantation. The posterior vitreous was visualized with triamcinolone and removed with core vitrectomy. Indocyanine green (ICG, 1.25 mg/mL, Eisai, Inc., Shenyang, China) was injected into the vitreous cavity to stain the ILM around the IMH, which was then peeled off from the inferior and temporal sides in a long strip shape approximately one diameter width of the optic disc, ensuring complete removal of the ILM at the edge of the IMH. To prepare for future scanning electron microscopy (SEM) examinations, the peeled‐off ILMs were harvested and placed into 4% formaldehyde and then stored at 4°C overnight for fixation. Considering the gravity, we grasped and peeled a flap from the superior and the temporal edge of residual ILM near the vascular arch, with an area of at least 2 papilla diameters (PDs). The pedicled ILM flap was attached to the optic disc and rotated to cover the IMH directly in a positive way. ABC was obtained from the patient’s elbow vein using a 2‐mL syringe, and then, it was immediately applied to fasten the ILM flap, followed by the gas–fluid exchange. Finally, the vitreous cavity was filled with 10% C3F8 as tamponade. All patients were required to keep a prone position for 7–14 days [6].

Figure 1Main process diagram. (a) The stained internal limiting membrane around the idiopathic macular hole was approximately peeled off. (b) Grasped and peeled a flap from the superior and the temporal edge of residual internal limiting membrane near the vascular arch, with an area of at least 2PD. (c) The internal limiting membrane flap is pedicled which attached to the macular hole. (d) Autologous blood clot was applied to fasten the internal limiting membrane flap, followed by the gas–fluid exchange.

**Figure:**
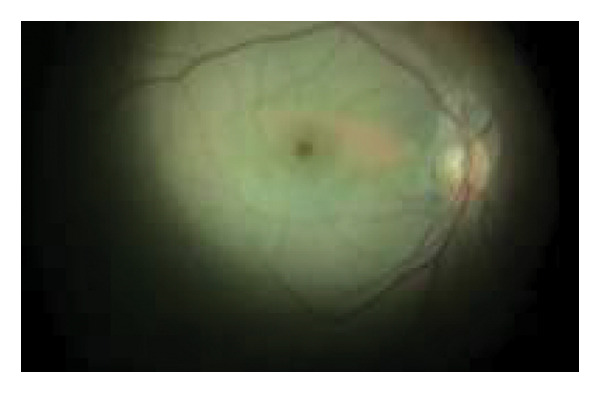
(a)

**Figure:**
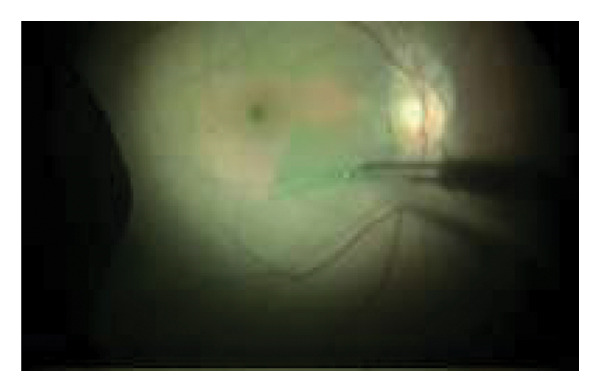
(b)

**Figure:**
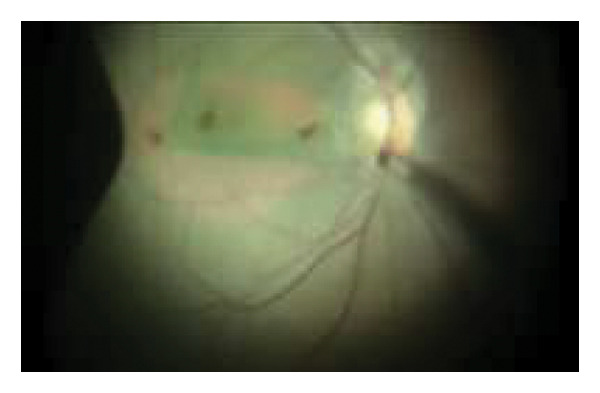
(c)

**Figure:**
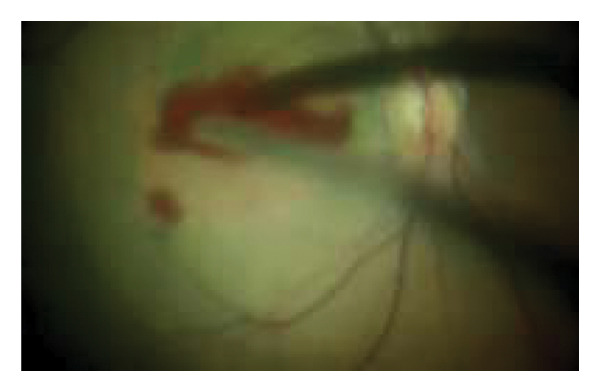
(d)

Referring to the protocol of SEM, the fixation of the membranes was done for a duration of 2 h using 2% glutaraldehyde in a buffer solution containing 0.1 M sodium cacodylate. Subsequently, the specimens underwent dehydration through a series of ascending ethanol solutions for 2 h. Preservation of the terminal structure was achieved by critical point drying with carbon dioxide (CPD 30, BAL‐TEC AG, Balzers, Liechtenstein) and followed by gold sputtering (SCD 005, BAL‐TEC AG). Finally, examination of the membranes was conducted using a LEO 1450 VP SEM (Carl Zeiss AG, Oberkochen, Germany).

Statistical analysis was conducted using SPSS software (Version 26.0, IBM SPSS Statistics). Normality of the data was tested using the Shapiro–Wilk test. To compare the data before and after the operation, the paired sample *t*‐test was utilized for analyzing the difference, assuming normal distribution of the data. In cases where the data did not follow a normal distribution, the paired Wilcoxon signed‐rank test was employed for comparison.

## 3. Results

**Figure 2:**
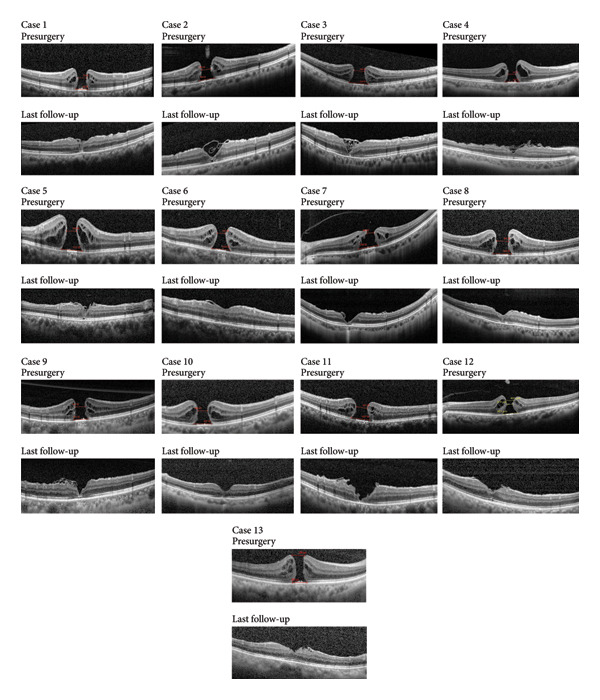
The preoperative and last follow‐up SD‐OCT images of 13 cases. All cases obtain macular hole closure.

All patients began the postsurgery follow‐up at 1 month, which lasted for a minimum of 3 months. The status of IMH closure or remaining open was confirmed using SD‐OCT images obtained at the final follow‐up. Restoration of foveal closure after IMH closure was categorized into U‐shape, V‐shape, W‐shape, and flap‐closure outcomes. The positive ILM rotational covering technique was successfully performed on 13 patients, resulting in IMH closure in all cases (100%) after a single procedure. Additionally, all patients experienced a significant improvement in BCVA compared to their preoperative vision. The mean IMH diameter and base aperture were 524.54 ± 85.96 μm and 992.69 ± 153.92 μm, respectively, as detailed in Table 1. At the last postoperative follow‐up, we discovered 7 eyes with U‐shape closure and 6 eyes with V‐shape closure. The flap closure was not observed in this study. All cases of IMH closure are illustrated in Figure 2.

**Table 1:** Preoperative basic data of patients with large idiopathic macular hole.

Case no.	Age	Sex	Eye	MLD (μm)	BD (μm)	Intraocular tamponade	Hole closure type	BCVA (logMAR)		Follow‐up, months	Complications	Duration of decreased vision, months
								Pre	Post			
1	64	F	Left	534	822	C3F8	V	1.00	0.60	7	None	8
2	68	F	Right	588	950	C3F8	V	1.00	0.70	26	None	1
3	71	F	Right	662	1246	C3F8	V	1.30	0.52	24	None	1
4	66	F	Right	441	897	C3F8	U	1.52	0.70	32	None	24
5	59	F	Left	545	1131	C3F8	U	1.22	0.60	29	None	3
6	66	M	Left	442	1034	C3F8	U	1.00	0.22	26	None	6
7	57	F	Left	450	1299	C3F8	V	1.30	0.30	24	None	8
8	68	F	Left	587	929	C3F8	U	1.22	0.60	13	None	24
9	60	F	Left	617	914	C3F8	V	0.82	0.60	3	None	10
10	61	F	Right	499	937	C3F8	U	1.30	0.92	15	None	2
11	61	F	Left	621	1057	C3F8	V	1.22	0.89	15	None	1
12	66	M	Left	408	889	C3F8	U	1.5	0.40	11	None	5
13	67	F	Right	425	800	C3F8	U	1.0	0.52	6	None	6
Overall	64.15 ± 4.18	F(11/13)	Left(8/13)	524.54 ± 85.96	992.69 ± 153.92	13	U/V = 7/6	1.18 ± 0.209	0.58 ± 0.202	17.77 ± 9.567	0	7.62 ± 7.848

*Note:* F: female; M: male.

Abbreviations: BCVA, best‐corrected visual acuity; BD, base diameter; MH, macular hole; MLD, minimum linear diameter.

The mean BCVA (logMAR) before surgery was 1.18 ± 0.209 and after surgery was 0.58 ± 0.202, showing a statistically significant difference (*p* < 0.001). The average follow‐up period was 17.77 ± 9.567 months. Macular foveal images were obtained using OCT 1 month after the operation due to C3F8 absorption. The horizontal and vertical deformations after operation were significantly improved compared with those before operation (*p* = 0.001, *p* < 0.001). The result of ERG analysis revealed that the response density in the postoperative period was improved, but the difference was not statistically significant (Table 2). The diameter of the defect of external limiting membrane (ELM) and ellipsoid zone (EZ) was significantly smaller at the 1‐month postoperative observation time (*p* < 0.001). And the defect size decreased further at the 3‐month follow‐up compared to the 1‐month follow‐up (ELM: *p* = 0.002; EZ: *p* < 0.001) (Table 3). No surgery‐related complications were reported during the follow‐up period.

**Table 2:** Functional changes pre‐ and postoperation.

Item	Preoperation	3 months postoperation	*p*
Vertical M‐score	0.45 ± 0.307	0.14 ± 0.767	0.001
Horizontal M‐score	0.58 ± 0.358	0.20 ± 0.147	< 0.001
BCVA (logMAR)	1.18 ± 0.209	0.58 ± 0.202	< 0.001
mf‐ERG R1 (8 cases)	78.29 ± 32.697	94.88 ± 37.374	0.161

*Note:* M‐score: the deformation score by M‐CHARTS. R: ring. Data are expressed as means ± standard deviation.

Abbreviations: BCVA, best‐corrected visual acuity; mf‐ERG, multifocal electroretinogram.

**Table 3:** The diameter changes in the ELM and EZ (pre‐ and postoperation) mean ± SD, μm.

Time point	ELM	EZ
Preoperation	1031.00 ± 224.49	1381.69 ± 270.31
1 month postoperation	386.54 ± 265.58	632.46 ± 264.69
*p*	< 0.001	< 0.001
3 months postoperation	214.08 ± 266.05	371.38 ± 285.97
*p*	< 0.001^∗^; 0.002^∗∗^	< 0.001^∗^;< 0.001^∗∗^

*Note:* *p* < 0.05 is statistically significant.

Abbreviations: ELM, external limiting membrane; EZ, ellipsoid zone.

^∗^Compared the 3‐month postoperation results with the preoperation results.

^∗∗^Compared the 3‐month postoperation results with the 1‐month postoperation results.

Surface examinations using SEM revealed smooth vitreal surfaces and rough retinal surfaces with the residual Müller cell debris, as illustrated in Figure 3.

Figure 3The vitreum (a) and retinal (b) surface of the internal limiting membrane in scanning electron microscopy.

**Figure:**
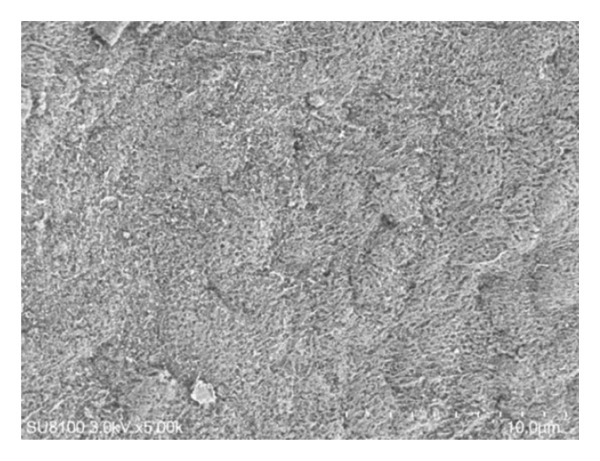
(a)

**Figure:**
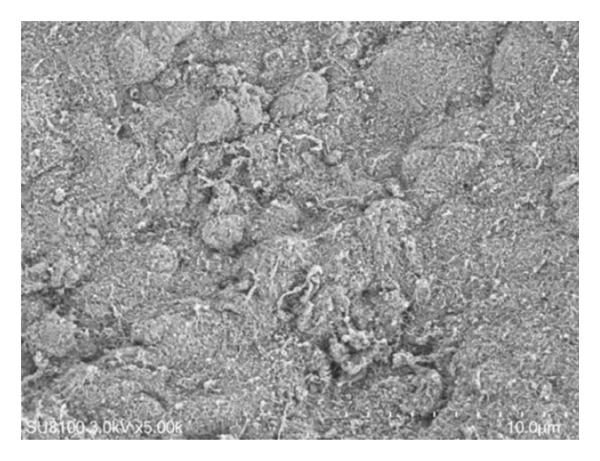
(b)

## 4. Discussion

IMH is a well‐known retinal disease classified by Gass in 1995 [7]. PPV combined with ILM peeling and gas tamponade, initially proposed by Kelly, has now emerged as the standard technique for treating IMHs [1]. Nonetheless, when dealing with large or recurrent MHs, the efficacy of the ILM peeling approach can be limited. This may be attributed to the fact that ILM peeling only focuses on the anteroposterior traction toward the fovea and the tangential tension of the ILM, without fully stimulating gliosis in the macular hole area.

Therefore, the ILM flap covering technique, introduced by Michalewska, utilizes the ILM flap as a scaffold for cell proliferation to aid in IMH recovery [3]. Numerous studies have shown that this technique can improve closure rates, particularly in cases of large IMHs (> 400 μm) [8–10]. Since then, various improvements have been made in the ILM flap covering technique, all of which have shown positive outcomes [11–14]. Additionally, alternative approaches such as ILM insertion, amniotic membrane transplantation, lens capsule transplantation, and neural retinal transplantation have been explored for treating large IMHs, but these methods have shown lower therapeutic effectiveness when compared to the ILM flap covering technique [15–18]. Along with the further research about the histopathological characteristics of ILM through an electron microscope, we could observe the difference between the vitreal side and retinal side. The vitreal side is smooth, whereas the retinal side is undulated, and the residual Müller cell debris could be detected on the retinal side. This result is consistent with the results of previous studies [19, 20].

Thus, the cover of the ILM retinal side over the IMH would theoretically supply a better structure for IMH closure. Besides, in our previous study [14], the inverted ILM flap technique resulted in only one “U‐shape” IMH closure out of 13 cases, whereas this new positive ILM flap technique led to “U‐shape” closures in more cases (7 out of 13). The successful repair of IMHs can be classified by OCT into two types: closure without neurosensory retinal defect and closure with neurosensory retinal defect (W‐shape). Based on the contour of the macular fovea on OCT, closure without neurosensory retinal defect can also be categorized as normal foveal contour (U‐shape) and step foveal contour (V‐shape). Postoperative visual acuity was best with U‐shape closure, and W‐shape closure was associated with the worst visual outcomes [21, 22].

Several previous articles have discussed the use of the positive ILM flap technique for treating IMHs, all demonstrating high rates of IMH closure and improved visual acuity. Tian proposed a surgical approach involving ILM peeling and in situ covering around the IMH to maintain retinal integrity [23]. However, this method may not be suitable for patients with IMHs larger than 400 μm. In contrast, Hu developed a new technique involving a pedicled ILM flap for IMHs in a positive way, which was flattened using PFCL [24]. This method may present challenges with the PFCL residue. Additionally, silicon oil was applied in some cases to prevent displacement of ILM, which required a second operation to remove the silicon oil. Our procedure differs from Hu’s in that we select the optic disc as the anchor point for the ILM flap. This technique effectively alleviates the tension on the temporal ILM of the IMH, which is a key factor in IMH formation [25].

Therefore, a modified method we invented is to rotate the pedicled ILM flap in a positive way from the upper vascular arch to cover the IMH following the complete ILM peeling around the IMH. The procedure involved peeling the ILM around the IMH from the inferior and temporal sides in a long strip shape approximately one diameter width of the optic disc. Subsequently, a 2‐PD‐wide ILM flap attached to the optic disc was directly rotated to cover the IMH. ABC was used to facilitate the flap adherence and 10% C3F8 was injected to fill the eye. This technique allowed for the release of tractive forces and ensured the creation of a sufficiently large ILM flap to cover the IMH, regardless of the condition of the ILM in the macular. As a result, all patients achieved closure of their IMHs and experienced a significant improvement in BCVA. According to the images of OCT in the last follow‐up, all patients presented U‐ or V‐shape, with no W‐shape detected. It is a better result compared to our previous study about inverted flap technique [14]. In our study, we observed a significant reduction in the extent of damage to the ELM and EZ of patients at 1 and 3 months postsurgery compared to presurgery. And the defect size decreased further at the 3‐month follow‐up compared to the 1‐month follow‐up. Interestingly, the degree of restoration of EZ was found to be lower than that of ELM, and patients with an intact EZ at the final follow‐up also maintained an intact ELM layer. This indicates that ELM may be the initial structure to recover following surgery for IMHs, which aligns with prior research findings [26]. The use of two‐line type M‐CHARTS effectively quantified metamorphopsia in patients with IMHs pre‐ and postsurgery [27, 28]. Specifically, our analysis using M‐CHARTS revealed a significant improvement in visual distortion in both vertical and horizontal directions at 3 months after surgery. There was an increasing trend of mf‐ERG, but it was not statistically significant, which may be due to the small number of cases.

The results of visual acuity recovery in our study were compared with previous research on the ILM flap technique. Compared to the visual acuity recovery associated with the inverted flap technique in our prior study [14], we observed a notable degree of improvement with this method. Additionally, there was an enhancement over the other positive methods [25]. However, when compared to Tian’s study [29], our improvement in visual acuity did not reach the same level. This discrepancy may be attributed to the smaller MH diameter reported in Tian’s cases (367.0 ± 120.7 μm) compared to our findings (minimum linear diameter: 524.54 ± 85.96 μm). Additionally, the baseline vision of participants in Tian’s study was superior to that of our participants.

Nevertheless, while our study identified a greater number of U‐shaped closures, we cannot definitively conclude that this technique surpasses the previous inverted flap method. A randomized controlled trial will be beneficial for verifying this hypothesis. Furthermore, additional mf‐ERG results are still needed to demonstrate the changes in retinal sensitivity of the macula following surgery.

In conclusion, the ILM rotational covering surgery with positive flap could provide sufficient ILM flap to cover the IMH in a physiological way. This approach proves to be a valuable treatment for larger IMHs (> 400 μm). The results of this study suggested that this technique may be helpful for anatomical and functional recovery. However, further studies with larger sample sizes and longer follow‐ups are required to confirm the efficacy of this approach. Prospective randomized controlled trials are also crucial to establish the superiority of the positive ILM flap technique.

**Nomenclature**
IMH: Idiopathic macular hole
PD: Papilla diameter
SEM: Scanning electron microscopy
BCVA: Best‐corrected visual acuity
EZ: Ellipsoid zone
ELM: External limiting membrane
ILM: Internal limiting membrane
ERM: Epiretinal membrane
SD‐OCT: Spectral domain optical coherence tomography
IOP: Intraocular pressure
ERG: Multifocal electroretinogram
PPV: Pars plana vitrectomy

## Ethics Statement

This study was approved by the Institutional Review Board of Xuzhou First People’s Hospital (Ethical approval number: xyyll[2021]‐XJSFX‐058). All methods were conducted in accordance with the tenets of the Declaration of Helsinki.

## Consent

All data were obtained with written informed consent from participants for participation in the study.

Written informed consent was obtained from all study participants.

## Disclosure

The funders had no role in study design, data collection and analysis, decision to publish, or preparation of the manuscript. All authors read and approved the final manuscript.

## Conflicts of Interest

The authors declare no conflicts of interest.

## Author Contributions

Haiyang Liu generalized the idea of the new technique, performed the surgery, and revised the manuscript. Huiyu Xi drafted the manuscript and interpreted the results. Yingying Song and Yewen Ni analyzed the patient data and performed the SEM examinations. Yumei Cao and Tianyu Zhu performed the OCT, mf‐ERG and M‐CHART examinations. Wei Fan edited the photos. Huiyu Xi, Yingying Song, and Yewen Ni contributed equally to this work and should be considered co‐first authors.

## Funding

This study was supported by Xuzhou Medical Key Talents Project (no. XWRCHT20220048), Xuzhou Key R & D Program (no. KC22099), and Natural Science Foundation of Jiangsu Province (BK20241765).

## Supporting Information

The video depicts the surgical procedure: firstly, the stained internal limiting membrane around the idiopathic macular hole was approximately peeled off. Secondly, a flap from superior and temporal edges of the residual internal limiting membrane near the vascular arch was grasped and peeled, with an area ≥ 2 PD. Subsequently, the internal limiting membrane flap is pedicled which attached to the macular hole. Finally, autologous blood clot was applied to fasten the internal limiting membrane flap, followed by the gas–fluid exchange.

## Supporting information


**Supporting Information** Additional supporting information can be found online in the Supporting Information section.

## Data Availability

The datasets used and analyzed during the current study are available from the corresponding author on reasonable request.
